# Delivery mode is a larger determinant of infant gut microbiome composition at 6 weeks than exposure to peripartum antibiotics

**DOI:** 10.1099/mgen.0.001269

**Published:** 2024-07-12

**Authors:** Sophie M. Leech, Danielle J. Borg, Kym M. Rae, Sailesh Kumar, Vicki L. Clifton, Marloes Dekker Nitert

**Affiliations:** 1 School of Chemistry and Molecular Biosciences, The University of Queensland, St Lucia, QLD, Australia; 2 Pregnancy and Development Group, Mater Research Institute, South Brisbane, QLD, Australia; 3 Faculty of Medicine, The University of Queensland, St Lucia, QLD, Australia; 4 Indigenous Health Group, Mater Research Institute, South Brisbane, QLD, Australia; 5 Mater Mothers’ Hospital, Brisbane, QLD, Australia

**Keywords:** antibiotics, birth, delivery mode, gut microbiome, infant, maternal

## Abstract

**Background.** Previous research has shown that delivery mode can shape infant gut microbiome composition. However, mothers delivering by caesarean section routinely receive prophylactic antibiotics prior to delivery, resulting in antibiotic exposure to the infant via the placenta. Previously, only a small number of studies have examined the effect of delivery mode versus antibiotic exposure on the infant gut microbiome with mixed findings.

**Objective.** We aimed to determine the effect of delivery mode compared to antibiotic use during labour and delivery on the infant and maternal gut microbiome at 6 weeks post-partum.

**Methodology.** Twenty-five mother–infant dyads were selected from the longitudinal Queensland Family Cohort Study. The selected dyads comprised nine vaginally delivered infants without antibiotics, seven vaginally delivered infants exposed to antibiotics and nine infants born by caesarean section with routine maternal prophylactic antibiotics. Shotgun-metagenomic sequencing of DNA from stool samples collected at 6 weeks post-partum from mother and infant was used to assess microbiome composition.

**Results.** Caesarean section infants exhibited decreases in *Bacteroidetes* (ANCOM-BC *q*<0.0001, MaAsLin 2 *q*=0.041), changes to several functional pathways and altered beta diversity (*R*^2^=0.056, *P=*0.029), while minimal differences due to antibiotic exposure were detected. For mothers, caesarean delivery (*P=*0.0007) and antibiotic use (*P*=0.016) decreased the evenness of the gut microbiome at 6 weeks post-partum without changing beta diversity. Several taxa in the maternal microbiome were altered in association with antibiotic use, with few differentially abundant taxa associated with delivery mode.

**Conclusion.** For infants, delivery mode appears to have a larger effect on gut microbiome composition at 6 weeks post-partum than intrapartum antibiotic exposure. For mothers, both delivery mode and intrapartum antibiotic use have a small effect on gut microbiome composition at 6 weeks post-partum.

## Data Summary

Raw sequence files with human DNA removed have been deposited in he Sequence Read Archive: BioProject accession: PRJNA1076911 which can be accessed at: https://www.ncbi.nlm.nih.gov/bioproject/?term=PRJNA1076911.

**Impact Statement**

The role of confounding intrapartum antibiotic use on infant gut microbiome dysbiosis associated with caesarean delivery was previously unclear. In this study we demonstrate that delivery mode itself is a larger determinant for infant gut microbiome composition than exposure to maternal peripartum antibiotics. However, both delivery mode and antibiotic use may influence maternal gut microbiota composition.

## Introduction

Colonization of the gut microbiome is hypothesized to begin at birth [1,3], and is influenced by factors such as delivery mode [4], breastfeeding and antibiotic use [5,7]. Dysbiosis of the gut microbiome in infancy is thought to affect future health outcomes including increased risk of asthma [8,9], atopy and obesity [6,10]. Caesarean section-associated dysbiosis is understood to be due to lack of exposure to maternal vaginal and faecal microbes during delivery, with infants delivered by caesarean section having an early microbiome resembling that of their mother’s skin [11], with ongoing depletions in bacteria such as *Bacteroides* and *Bifidobacterium* [4,12, 13], which are the dominant genera in vaginally delivered, breastfed infants [4,14].

However, many hospitals routinely administer intravenous antibiotics prior to caesarean section, resulting in antibiotic exposure to the infant via the placenta just prior to birth [15]. This exposure may play a role in the dysbiosis associated with caesarean delivery [16]. In addition, the maternal vaginal microbiota is reportedly not predictive of the infant faecal microbiome, suggesting that lack of exposure to vaginal microbes is unlikely to be the cause of the differences between vaginally and caesarean-delivered infants [17]. Furthermore, vaginal seeding cannot fully restore the infant gut microbiota of caesarean-delivered infants [18,19], though exposure to maternal faecal matter appeared to restore gut microbiota composition in one study [20].

Previous attempts to distinguish the effects of maternal antibiotics from caesarean section on the infant gut microbiome using 16S rRNA gene amplicon sequencing methods have had mixed results. Delaying antibiotic exposure to after cord clamping in caesarean deliveries was still associated with decreases in the key bacterial taxa *Bifidobacterium* and *Bacteroides* [12,21]. In contrast, *Bifidobacterium* abundance decreased with maternal antibiotic use during delivery but was unaffected by delivery mode, while depletions in *Bacteroides* were dependent on delivery mode, but unaffected by antibiotic use [7].

Therefore, we aimed to further investigate the effects of maternal antibiotic use and delivery mode using shotgun metagenomic sequencing. We analysed 25 mother–infant dyads to evaluate the effect of caesarean section and antibiotic use on the infant gut microbiome composition and function, including antibiotic resistance gene abundance, and the relationship with the maternal microbiome at 6 weeks post-partum.

## Methods

### Sample selection

Twenty-five mother–infant dyads were selected from the Queensland Family Cohort (QFC) pilot study [22] including nine vaginally delivered infants without antibiotic exposure (VnAB), seven vaginally delivered infants with maternal intrapartum antibiotic use (VAB) and nine caesarean-delivered infants with routine maternal intrapartum antibiotic use (CS). Vaginal delivery (VD) included instrumental, non-instrumental and water births. CS included scheduled and emergency caesarean sections, with IV antibiotics administered immediately prior to the procedure.

Dyads were selected based on singleton pregnancy; hospital birth after 36+0 weeks of gestation; 10–90th percentile for birthweight; maternal pre-pregnancy body mass index 18.3–30 kg m^–2^; breastfeeding, and unaffected by (pre)eclampsia, (gestational) diabetes mellitus, infant antibiotic or probiotic use, maternal antibiotic use between 24 weeks of gestation and 6 weeks post-partum (excluding during labour and delivery for CS and VAB) or disease that could potentially affect the microbiota or infant growth. A roughly equal number of male and female infants were selected [male infants (%): VnAB *n*=5 (55.6), VAB *n*=3 (42.8), CS=4 (44.4)]. For more details of the sample selection process refer to Fig. S1 (available in the online version of this article, Supplementary Material 1).

All infants were breastfed with ten infants receiving supplementary formula during the first 6 weeks of life.

### Collection of stool samples

Samples were collected by the mother at 6 weeks post-partum using a sterile FLOQSwab-ADT (Microba) [23] on toilet paper (maternal) or from a nappy (infant). Swabs were dried at room temperature for at least 24 h and then stored at −20 °C.

### Extraction of DNA from stool swabs

Different kits were used for extraction of DNA due to the nature of study expansion and kit availability at the time of the study. Samples from five mother–infant dyads were processed by Microba (Brisbane, Australia) with the QIAamp 96 PowerFecal QIAcube HT kit (Qiagen) to extract DNA. The remaining swabs were isolated using the QIAamp Fast DNA Stool kit (Qiagen) with a modified protocol to include a bead beating step (detailed below; ten dyads), or the QIAamp PowerFecal Pro DNA kit (Qiagen) according to the manufacturer’s protocol (ten dyads). This does introduce the risk of batch effects, which were tested for at each stage of analysis.

#### Bead beating step

To extract DNA from faecal swabs, swabs were submerged in 600 µl of InhibitEX buffer and bead beaten in a TissueLyser (Qiagen) with a mixture of 0.1 and 0.5 mm Zirconia beads for 5 min at 30 Hz. The supernatant was transferred to a new tube and the standard protocol for the kit was followed. The QIAamp PowerFecal Pro DNA kit contains an in-built bead beating step.

### Metagenomic sequencing and quality control

Shotgun metagenomic sequencing was performed with NovaSeq6000 (Illumina) using 2×150 bp paired-end chemistry as a fee for service by Microba (*n*=5 mother–infant dyads), and Centre for Microbiome Research (Brisbane, Australia) (CMR) (*n*=19 mother–infant dyads) with a target depth of 3 Gb. Library preparation was performed with the Illumina DNA Prep kit (Illumina #20018705).

Quality control of sequences was conducted using the Galaxy platform [24]. The tools ‘FastQC Galaxy version 0.73+galaxy0’ [25], ‘MultiQC Galaxy version 1.11+galaxy0’ [26], ‘Trimmomatic Galaxy version 0.36.6’ with inclusion of an initial Illumina clip step with Nextera (paired-end) sequences [27], ‘Bowtie 2 Galaxy version 2.4.2+galaxy0’ with reference genome, *Homo sapiens* hg38, for removal of host sequences [28], and ‘Samtools view Galaxy version 2.9+galaxy3’ and ‘Samtools fastx Galaxy version 1.9+galaxy1’ [29] were used with default settings. On average 2.22% (range 1.59–7.66)%) of reads were removed during quality control due to being of host origin from maternal samples and 1.53±0.59 % from infant samples.

Four maternal samples were excluded due to insufficient reads post-quality control [3 033 257 (998 311 – 5 311 849) reads]. These samples clustered separately to the other maternal samples (Supplementary Material 1 Fig. S2) and were thus discarded. This reduced maternal numbers to *n*=8 VnAB, *n*=6 CS and *n*=7 VAB. Remaining infant and maternal samples were then randomly rarefied to equal the minimum depth sample (10 603 798 reads per sample) using ‘seqtk’ [30] prior to alpha and beta diversity analysis and before analysis with ‘MaAsLin2’ [31]. As ‘ANCOM-BC’ includes a step adjusting for sequencing depth and expects raw counts as inputs, samples were not rarefied prior to analysis with ‘ANCOM-BC’ [32].

### Taxonomy profile, gene pathway analysis and detection of antimicrobial resistance genes

‘MetaPhlAn4’ (version 4.0.3) [33] and ‘HUMAnN 3.6’ [34] were used to generate taxonomic and functional profiles, respectively, with default settings. For identification of antimicrobial resistance genes, paired-end sequences were input into the ‘metaSPAdes’ [35] metagenome assembler (Galaxy version 3.15.4+galaxy2) using the Galaxy Australia online platform with automatic k-mer detection and automatic Phred quality offset to assemble contigs. Resistance gene identifier (version 6.0.2) with ‘CARD’ database (version 3.2.7) [36] was used to identify resistance genes in contigs with the option for low-quality assemblies enabled to allow for inclusion of partial genes.

### Statistical analysis

Analysis was largely conducted using ‘RStudio’ (R version 4.0.5) [37]. For beta diversity analysis, the packages ‘mixOmics’ [38], ‘phyloseq’ [39] and ‘VEGAN’ [40] were used. Prior to beta diversity analysis of composition, a ‘pseudo-count’ of 0.0001 % was applied followed by centred-log ratio transformation (CLR). Pathway abundance was normalized to copies per million (CPM) and a pseudo-count of 1 was applied prior to CLR transformation before principal components analysis (PCA).

Statistical analysis of beta diversity was conducted with adonis2 (VEGAN [40]) with 9999 permutations using a Euclidean distance matrix from CLR-transformed data for delivery mode, maternal antibiotic use and batch with setting ‘by=margin’ for infants and mothers. Distance between unrelated and related mother–infant dyads, and by delivery mode and antibiotic use status was calculated from the Euclidean distance matrix from CLR-transformed data in R and analysed in GraphPad Prism 9.0 using either an unpaired t-test, ANOVA or Mann–Whitney test for normally distributed and non-normally distributed data, respectively. Data was treated as normal if it passed the Shapiro–Wilk test, the Kolmogorov–Smirnov test, D’Agostino and Pearson test, and the Anderson–Darling test (GraphPad prism). Data is presented as mean±sd if normally distributed or median (interquartile range, IQR) if non-normally distributed.

Supervised analysis was also conducted using sPLS-DA (sparse partial least squares discriminant analysis) in ‘mixOmics’ [38] for combined delivery mode–antibiotic use group, delivery mode alone, antibiotics alone, and batch with scaling and centring set to false. Alpha diversity was evaluated using the Shannon Diversity index, Richness and Evenness in R.

Differential abundance analysis was conducted using ‘ANCOM-BC’ [32] and ‘MaAsLin2’ [31]. The input for ‘MaAsLin2’ was relative abundances with an arcsine square-root transformation applied with a minimum abundance threshold of 0.0001 and minimum prevalence threshold of 0.2. The input for ‘ANCOM-BC’ was approximate counts calculated by multiplying sequencing depth by relative abundance with a 0.2 prevalence threshold. The *q*-values are adjusted *P*-values using the Benjamini–Hochberg procedure (MaAsLin2) or Holm–Bonferroni method (ANCOM-BC). Both MaAsLin2 and ANCOM-BC only allow for comparison of a single reference group with every other group. Therefore, for batch, MaAslin2 and ANCOM-BC analysis was repeated with an alternate reference group to allow for comparisons between all three groups. The *q*-values were then recalculated after adding the additional batch comparisons using the R function ‘p.adjust’.

For antimicrobial resistance gene analysis, heatmap2 from the package ‘gplots’ [41] was used with clustering based on a distance matrix calculated using the ‘dist’ function with method ‘binary’. Both ‘Perfect’ and ‘Strict’ hits identified by RGI were treated as present genes.

## Results

### Participant characteristics

There were differences in maternal age (*P=*0.05), gravidity by antibiotic use (*P=*0.013), antibiotic type (*P*=0.0068) and reason for antibiotics (*P=*0.0063) between groups (Tables 1 and Supplementary Material 1 Table S1). CS mothers mainly received antibiotics due to surgery prophylaxis (*n*=9/9) and VAB mothers for premature rupture of membranes (PROMs; *n*=5/7) (Supplementary Material 1 Table S1). No infant characteristics were statistically significantly different. There were a relatively large number of missing values for parity and gravidity (9 out of 25), and hence results concerning these variables should be treated with caution.

**Table 1.** Maternal and infant characteristics nAB: no antibiotics during delivery, AB: antibiotics during delivery. Expanded characteristics are available in Table S1.

	Vaginal (nAB) (*n*=9)	Vaginal (AB) (*n*=7)	Caesarean (*n*=9)	***P*-value**
Maternal age (years)*	33 (29.5–34.5)	28 (24–30)	31 (30–36.5)	0.05
Maternal pre-pregnancy BMI (kg m^–2^)*	22.3±1.5	22.0±3.6	22.8±3.5	0.85
Nulliparous (parity=0)	2^6^	0^4^	0^6^	0.5/0.12†
Multigravida (1<gravidity)	1^6^	4^4^	4^6^	0.52/0.013
Emergency caesarean	–	–	5	
Gestational weight gain (pre-pregnancy to 36 weeks) (kg)	15.5±3.6	11.3±4.6	12.5±4.2^7^	0.13
Number with chronic disease	6	6	8	0.45
Affected by >1 chronic disease	3	4	4	0.64
Male (%)	5 (55.6)	3 (42.8)	4 (44.4)	0.85
Gestational age at delivery (days)	277±6	276±6	270±9	0.12
Exclusively breastfed (%)	6 (66.7)	6 (85.7)	3 (33.3)	0.09
5 min Apgar score*	9 (9–9)	9 (9–9)	9 (9–9)	>0.99
**Ethnicity**				0.55
Caucasian	5	6	7	
Other	4	1	2	
**Antibiotic type**				0.0068
Ampicillin	–	2	0	0.062
Cefazolin	–	0	8	0.0014
Ampicillin and cefazolin	–	0	1	>0.99
Ampicillin, cefazolin and metronidazole	–	2	0	0.18
Benzylpenicillin	–	2	0	0.18
Unknown	–	1	0	0.44
**Infant measurements at birth**				
Birthweight (g)	3554±554	3238±265	3304±253	0.24
Body length (cm)	52.0±2.3	49.6±1.0	51.2±2.05	0.06
Head circumference (cm)*	35.0 (34.0–36.0)	35.0 (33.5–36)	36.0 (33.5–36.3)	0.56
Abdominal circumference (cm)*	32.0 (29.5–32.3)^5^	29.8 (28.0–32.4)^4^	30.5 (30.0–31.75)	0.68
Upper arm circumference (cm)	11.4±1.4^6^	10.4±1.0^5^	10.2±0.8	0.13
**Infant measurements at 6** **weeks**				
Weight (g)*	4650 (4375–5240)	4750 (4499–5025)	4400 (4370–4900)^7^	0.64
Body length (cm)*	57.0 (55.5–58.8)	56.0 (54.0–56.0)	54.0 (53.0–58.3)	0.08
Head circumference (cm)	38.0±1.1	38.4±0.84	38.2±0.9	0.65
Abdominal circumference (cm)*	38.0 (36.3–39.8)^8^	40.0 (38.0–40.9)^6^	38.0 (35.8–39.3)	0.23
Upper arm circumference (cm)*	12.3 (11.5–12.8)	12.8 (11.1–13.6)^6^	12.0 (11.5–12.8)	0.60

Data displayed as mean±sd, *median (IQR), 4*n*=4, 6*n*=6, 7*n*=7, 8*n*=8. †Due to missing values, chi-square calculation across three groups was not possible as test conditions were not met. Therefore, columns were combined for a delivery mode comparison and an antibiotic exposure comparison. *P*-values are presented in the form delivery mode/antibiotic exposure.

VnAB, VAB and CS were not equally distributed across the different sequencing batches (Supplementary Material 1 Table S1).

As this was the case, the effect of batch was evaluated but found to be unlikely to drive results. All results associated with batch are presented in the supplementary material (Supplementary Material 1 and Supplementary Material 2 Figs S3, S4G−I, S7 and S8F–H, Tables S2–7 and Discussion S1). While not significantly different across groups, more CS infants were mixed fed (Table 1). As breastfeeding vs formula feeding is known to be an important influencer of the infant gut microbiome [5], an analysis of exclusively breastfed vs mixed fed infants was also conducted to ensure there was no effect on the results. There was no effect of feeding on alpha or beta diversity (Supplementary Material 1 Figs S10 and S11) or on differential abundance of taxa or metabolic pathways.

### Infant gut microbiota

**Fig. 1.**
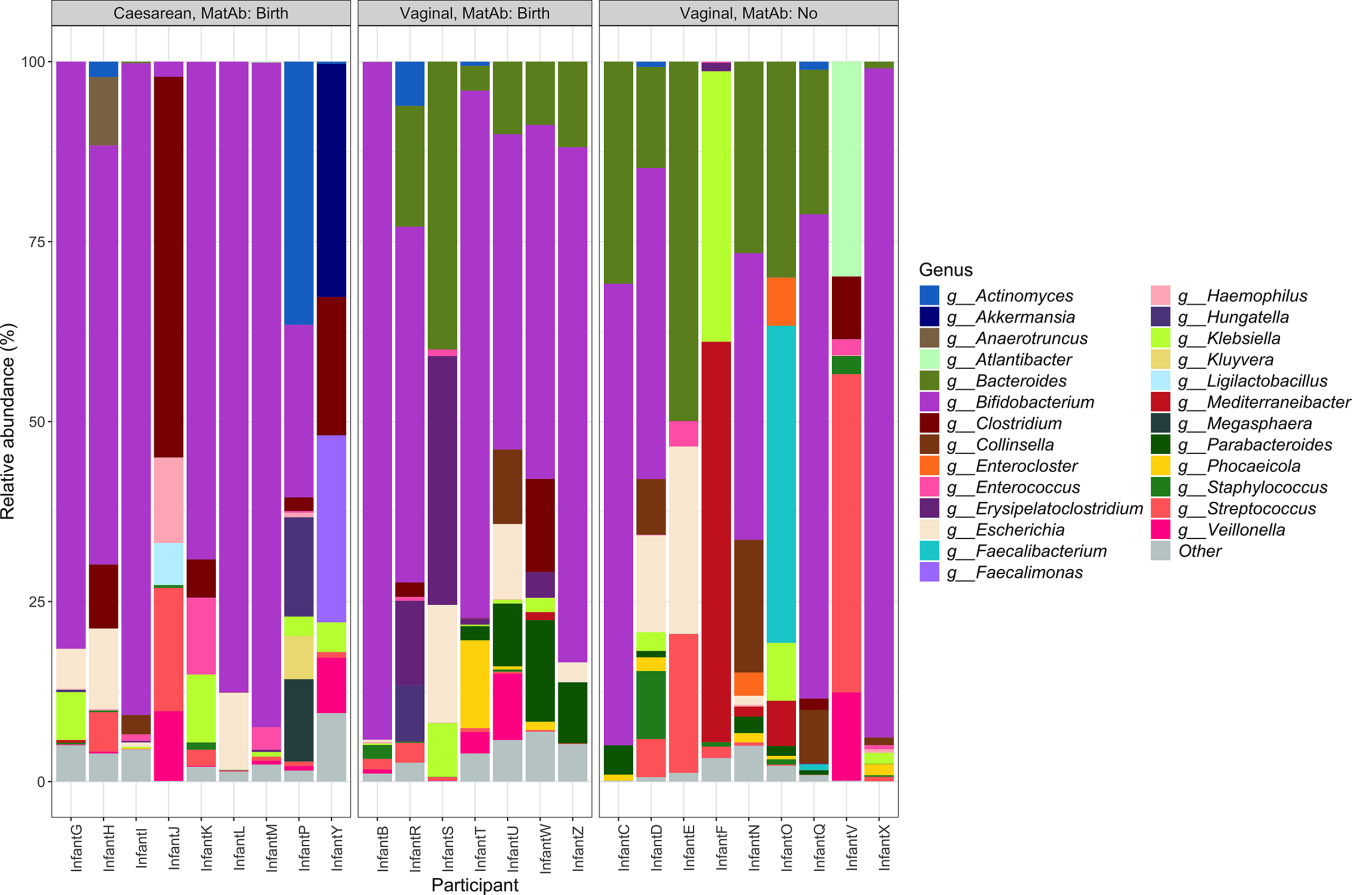
Composition of the infant gut microbiome at 6 weeks, presented at the genus level with ‘other’ representing taxa present at <5 % relative abundance in all samples. Data are grouped by delivery mode (vaginal or caesarean) and antibiotic use [antibiotics exposure during delivery (MatAb:Birth) or no exposure (MatAb: No)].

*Bifidobacterium* dominated most infant gut microbiome samples (Fig. 1). *Bacteroides* was also abundant in most vaginally delivered infant gut microbiomes, but was visibly absent from the gut microbiomes of caesarean-delivered infants (Fig. 1). Other genera frequently present at lower abundance include *Clostridium, Klebsiella, Streptococcus* and *Escherichia.*

**Fig. 2.**
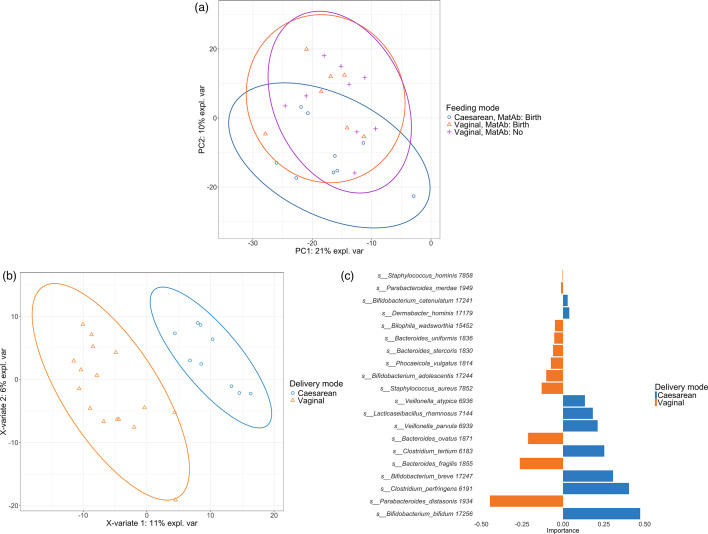
(a) Principal component analysis of infant gut microbiota coloured by delivery mode and maternal antibiotic use (MatAb) status. Ellipses represent 95 % confidence interval. (b) Sparse partial least squares linear regression for discriminant analysis (sPLS-DA) of infant gut microbiota separated by delivery mode with (c) taxon contributions to component 1.

Using PCA, delivery mode drives changes along PC2 which captures 10 % of the variation in the infant gut microbiota (adonis2 *R*^2^=0.056, *P*=0.029) (Fig. 2a), while maternal antibiotic use (adonis2 *R*^2^=0.046, *P*=0.20) did not influence infant gut microbiota beta diversity. There were no significant differences in alpha diversity (Supplementary Material 1 Fig. S4).

Abundance of the phylum *Bacteroidetes* was increased in VD infants as measured by ANCOM-BC (*q*<0.0001) and Maaslin2 (*q*=0.041). In addition, ANCOM-BC identified several significantly abundant taxa associated with delivery mode and antibiotics (Tables 2 and S2). Using sPLS-DA (Fig. 2b), decreases in *Bacteroidetes* species and increases in *Veillonella, Bifidobacterium* and *Clostridium* determined the separation of CS samples by delivery mode on component 1 (Fig. 2c).

**Table 2.** Significant differentially abundant species, genera and metabolic pathways (ANCOM-BC) in infants by delivery mode and antibiotics at the species and genus level VD: vaginal delivery, AB: antibiotics.

	Delivery mode	Antibiotics
**Species**
*Parabacteroides distasonis* SGB 1934	↑ VD (*q=*0.0003)	ns
*Clostridium tertium* SGB 6183	↓ VD (*q*=0.003)	ns
*Clostridium perfringens* SGB 6191	↓ VD (*q=*0.0009)	ns
*Dolosigranulum pigrum* SGB 7017	ns	↑ No AB (*q<*0.0001)
**Genus**		
*Bacteroides*	↑ VD (*q<*0.0001)	ns
*Phocaeicola*	↑ VD (*q=*0.03)	ns
*Parabacteroides*	↑ VD (*q=*0.0007)	ns
*Gemella*	↓ VD (*q*=0.009)	ns
*Dolosigranulum*	ns	↑ No AB (*q<*0.0001)
**Metabolicpathways**		
GALACT-GLUCUROCAT-PWY: superpathway of hexuronide and hexuronate degradation	↑ VD (*q=*0.0007)	ns
GLUCUROCAT-PWY: superpathway of β-d-glucuronoside degradation	↑ VD (*q=*0.001)	ns
GLUDEG-I-PWY: GABA shunt	↑ VD (*q=*0.01)	ns
PWY-7456: β-(1,4)-mannan degradation	↑ VD (*q<*0.0001)	ns

There were alterations in several significantly different pathways (Table 2 and Supplementary Material 1 Table S3) and reactions (Supplementary Material 2 Table S4) using ANCOM-BC associated with delivery mode. However, maternal intrapartum antibiotic use was not associated with altered functional pathways or with the presence of antimicrobial resistance genes in the gut microbiome of infants (Supplementary Material 1 Fig. S5A). These results suggest that mode of delivery is the main driver of the composition of the infant gut microbiota at 6 weeks post-partum.

### Maternal gut microbiota

**Fig. 3.**
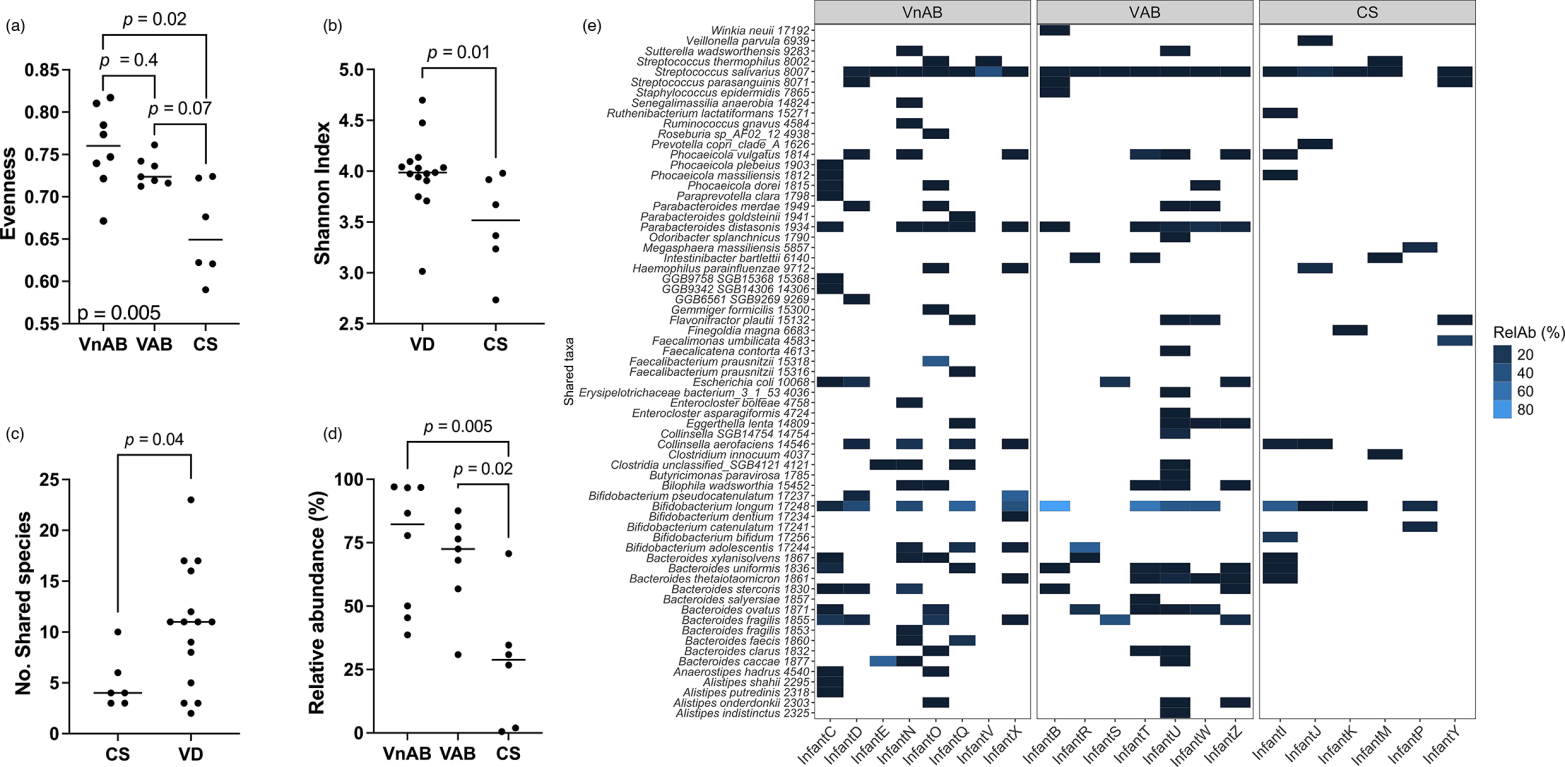
(a) Maternal gut microbiota diversity evenness by delivery mode and antibiotic use status. (**b**) Maternal Shannon index by delivery mode. (**c**) Shared species between related mother–infant dyads delivered by caesarean section (CS) and vaginal delivery (VD). (**d**) Relative abundance of shared species in infants delivered vaginally without antibiotics (VnAB), vaginally with antibiotics (VAB) and caesarean delivery (CS) (*P=*0.004). (**e**) Relative abundance (RelAb) of shared species in the infant gut microbiota (*n*=21).

While there were no significant differences in beta diversity in the maternal gut microbiome at 6 weeks post-partum (Supplementary Material 1 Figs S6 and S7), evenness was increased in women delivering vaginally (*P*=0.0007) and decreased with antibiotic use (*P*=0.016) (Supplementary Material 1 Fig. S8A, C). Interestingly, the VAB group, which delivered vaginally but were exposed to intrapartum antibiotics, forms an intermediate group between VnAB and CS, suggesting both delivery mode and antibiotic use affect maternal gut microbiota evenness at 6 weeks post-partum (Fig. 3a). There were no significant differences in richness and the differences in the Shannon index (*P=*0.014) are probably driven by the difference in evenness of the gut microbiome (Fig 3b and Supplementary Material 1 Fig S8).

Several taxonomic and functional changes (Table 3 and Supplementary Material 1 and Supplementary Material 2 Tables S5–S7) were detected associated with delivery mode and antibiotics with ANCOM-BC but not MaAsLin2 in the maternal gut microbiota. The presence of antimicrobial resistance genes was not affected by maternal antibiotic use during labour and delivery (Supplementary Material 1 Fig. S5B).

**Table 3.** Significant differentially abundant species, genera and metabolic pathways (ANCOM-BC) in mothers by delivery mode and antibiotics at the species and genus level VD: vaginal delivery, AB: antibiotics.

	Delivery mode	Antibiotics
**Species**
*Clostridia bacterium* SGB14861	↑ VD (*q=*0.0006)	ns
*Roseburia faecis* SGB4925	ns	↑ No AB (*q<*0.0001)
*Prevotella disiens* SGB1559	ns	↓ No AB (*q=*0.04)
*Butyricicoccus* sp. AM29 23AC SGB14991	ns	↑ No AB (*q<*0.0001)
*Blautia glucerasea* SGB4816	ns	↑ No AB (*q=*0.001)
*Lacrimispora celerecrescens* SGB4868	ns	↓No AB (*q=*0.007)
*Lachnospiraceae bacterium* SGB4750	ns	↑ No AB (*q<*0.0001)
*Clostridiaceae bacterium* SGB4721	ns	↓ No AB (*q=*0.045)
GGB4277 SGB5832	ns	↓ No AB (*q=*0.04)
**Genus**		
*Prevotella*	↓ VD (*q*=0.0002)	ns
*Intestinimonas*	↑ VD (*q=*0.04)	↓ No AB (*q=*0.007)
*Butyricicoccus*	ns	No AB (*q<*0.0001)
**Metabolicpathways**		
METHGLYUT-PWY: superpathway of methylglyoxal degradation	ns	↓ No AB (*q=*0.0002)
PWY-6906: chitin derivative degradation	↑ VD (*q=*0.01)	ns
PWY-7315: dTDP-*N*-acetylthomosamine biosynthesis	↑ VD (*q<*0.0001)	ns
PWY490-3: nitrate reduction VI	ns	↑ No AB (*q=*0.02)

### Shared species

CS infants shared fewer species with their mothers than VD infants (*P*=0.041) (Fig. 3c) and these shared species comprised a smaller proportion of their microbiome (VnAB-CS *P=*0.005, VAB-CS *P=*0.017) (Fig. 3d). However, maternal intrapartum antibiotic use did not affect the number of shared species between related mother–infant dyads (*P*=0.16) (Supplementary Material 1 Fig. S9G). The most frequently shared species between mothers and infants was *Streptococcus salivarius* (SGB8007), which was shared in 19 pairs (90.5 %) (Fig. 3e). Differences in the number of shared species appeared largely driven by absence of species such as *Parabacteroides distasonis* (SGB1934) and various *Bacteroides* species which tended to be shared amongst vaginally delivering dyads but absent amongst caesarean section infants (Fig. 3e).

## Discussion

Delivery mode, rather than exposure to maternal intrapartum antibiotics, is a greater driver of the composition of the infant gut microbiome at 6 weeks post-partum, in agreement with previous studies [12,21]. In addition, delivery mode and intrapartum antibiotic exposure caused significant changes to the maternal gut microbiome at 6 weeks post-partum.

Overall, the composition of the infant gut microbiome at 6 weeks was consistent with previous findings of infants at 6 weeks, with *Bacteroides* and *Bifidobacterium* dominating, and other taxa such as *Clostridium*, *Streptococcus* and members of *Enterobacteriaceae, Klebsiella* and *Escherichia* also being prevalent [42,44]. This is also consistent with findings in infants at similar time points [4,12]. The increase in the phylum *Bacteroidetes* in vaginally delivered infants appears to be driven by increases in species such as *Parabacteroides distasonis, Bacteroides fragilis* and *Bacteroides ovatus.* However, of these, only *P. distasonsis* was statistically significantly different. This aligns with other studies that have previously reported the genus *Bacteroides* to be decreased in caesarean-delivered infants [4,7, 12, 13, 21, 45], probably also driving the lower number of maternally shared species. Furthermore, in CS infants, *Clostridium tertium* SGB 6183 and *Clostridium perfringens* SGB 6191 are increased, which agrees with previous reports [21,46].

Previously *Bifidobacterium* has been reported to be decreased by antibiotic exposure [7], and by a caesarean delivery [12,13, 21, 46]. However, this was not observed in the present study. The previous reports largely used lower resolution 16S rRNA gene amplicon sequencing technology with major differences in *Bifidobacterium* seen before 6 weeks of age. Hence both difference in method and time point may contribute to the lack of differences in *Bifidobacterium* seen in the present study.

As we see a difference by delivery mode but not by antibiotic use, this suggests that the lack of exposure to rectal microbes upon exit of the birth canal [45] is likely the main driver of differences between caesarean-delivered and vaginally delivered infants. However, while we did examine shared species, to truly assess maternal–infant transmission, a greater sequencing depth to allow for strain analysis, along with sequential sampling at earlier time points, is required.

The functional capacity of the infant gut microbiota may also be altered by delivery mode. The β-(1,4)-mannan degradation pathway was increased in vaginally delivered infants. This pathway was reported to be increased in infants with a *Bacteroidaceae-*dominated gut microbiome [47], which is common in vaginally delivered infants. Interestingly, β-(1,4)-mannans are complex plant-based sugars, which breastfeeding infants are not exposed to prior to the introduction of solid foods [47]. Whether differing abundance of this pathway would impact tolerance and utility of these sugars upon the introduction of solid food is purely speculative, though differences in specific *Bacteroides* species even after the introduction of solid food have been previously reported [4].

Two pathways involved in the degradation of β-d-glucuronides were also more abundant in vaginally delivered infants. β-d-glucuronides are products of detoxification, with glucuronidation of molecules allowing for their excretion via the gastrointestinal tract or the kidneys [48]. In the first step of this pathway, bacterial β-glucuronidase reverses the inactivating glucuronidation, regenerating the active molecule [48,49]. Hence, bacterial β-glucuronidase may play a role in the regulation of endogenous compounds such as oestrogen and bilirubin [48,50], and in pharmacokinetics [48,49]. Whether an increase in this pathway would have implications for the health of vaginally delivered infants compared to caesarean-delivered infants is, however, unclear.

One pathway (GLUDEG-I-PWY) beginning with 2-oxoglutarate and ending with the production of succinate was more abundant in vaginally delivered infants. Previous work tracking the short-chain fatty acid profile over the first 2 years of life in breastfed, vaginally delivered infants noted high succinate in the first months after birth [51]. Succinate levels tended to be depleted in the stool of caesarean section-delivered infants at 6 weeks post-partum [52]. Unfortunately, no additional stool was available for analysis of succinate in the present study, and the impacts of succinate on health are not well studied compared to other short-chain fatty acids.

The maternal gut microbiome was affected by both intrapartum antibiotic use and delivery mode, with a reduced evenness of the microbiome at 6 weeks post-partum. Antibiotics are well known to influence gut microbiota composition with differences in richness seen in non-pregnant individuals up to 2 months post-treatment with specific differences in composition persisting even beyond this [53]. The cause of the difference associated with the delivery mode is, however, not entirely clear, though it does not seem to be fully explained by antibiotic use. Minimal research exists examining the effect of non-gastrointestinal surgery on the gut microbiota. In one study examining the gut microbiota following cardiac surgery, differences were attributed to iv antibiotics; however, the surgery itself may also have had direct effects [54]. We therefore speculate that inflammation after surgery, as well as differences in physical recovery times, and hence activity levels, and dietary patterns between caesarean section and vaginal deliveries, may be responsible for the differences we observed.

### Strengths and limitations

Differences in the type of antibiotics received and the reason for the antibiotic exposure may have affected results. Most women undergoing caesarean deliveries received cefazolin while the majority of those who delivered vaginally received ampicillin alone or in combination with cefazolin or metronidazole, or benzylpenicillin. While both cefazolin and ampicillin are broad-spectrum beta-lactam antibiotics, benzylpenicillin is a narrow-spectrum antibiotic targeting Gram-positive bacteria and thus may have a different effect on the gut microbiota [55]. However, only two women delivering vaginally were treated with benzylpenicillin alone, and hence this is unlikely to have a major effect on the results. Most women delivering vaginally received antibiotics for PROMs, rather than for routine surgical prophylaxis in caesarean section. A previous study found that PROMs altered the composition of the infant meconium but this disappeared 4–7 days after birth [56], so this is unlikely to be an important factor for infant gut composition at 6 weeks.

While shared species within dyads were assessed in this study, accurate assessments of vertical transmission are not possible without strain-level analysis, which was not available in this study due to insufficient sequencing depth and coverage of key species. Additionally, more information regarding colonisation of the infant gut could be gained by sampling additional time points before and after 6 weeks post-partum. While it is evident that delivery mode contributes to microbial dysbiosis in infancy, the health implications of this are not yet clear. Further research is required to determine the effects on long-term health and wellbeing. Finally, this study was limited by samply size, and an additional larger study is warranted to confirm the findings reported here.

## Conclusions

Overall, this study suggests that delivery mode itself is a greater driver of infant gut microbiome composition than intrapartum exposure to antibiotics, though antibiotics may still affect the abundance of some taxa. Beta diversity of the infant gut was significantly altered by delivery mode and *Bacteroidetes* was reduced in infants born by caesarean section. Further metagenomic sequencing analyses in a larger sample are needed to accurately identify changes in microbial species and metabolic capability due to delivery mode and antibiotic exposure on the infant gut microbiome.

## supplementary material

10.1099/mgen.0.001269 Supplementary Material 1.

10.1099/mgen.0.001269 Supplementary Material 2.

## Abbreviations

ANCOM-BC2: Analysis of compositions of microbiomes with bias correction 2
CLR: centred-log ratio transformation
CPM: copies per million
CS: caesarean-delivered infants with routine maternal intrapartum antibiotic use
IQR: interquartile range
MaAsLin 2: Microbiome Multivariable Associations with Linear Models 2
PCA: principal components analysis
PROMs: premature rupture of membranes
QFC: Queensland Family Cohort
SGB: Species-level genome bin
sPLS-DA: sparse partial least squares discriminant analysis
VAB: vaginally delivered infants with maternal intrapartum antibiotic use
VD: vaginal delivery
VnAB: vaginally delivered infants without antibiotic exposure
