# Supplementary material for: Delivery mode is a larger determinant of infant gut microbiome composition at 6 weeks than exposure to peripartum antibiotics
Source: Microb Genom. 2024 Jul 12;10(7):001269. doi: 10.1099/mgen.0.001269 (PMC11316550; doi:10.1099/mgen.0.001269)
Supplement: Supplementary Material 1. [file mgen-10-01269-s001.pdf]

Supplementary Discussion 1

Batch

This study was affected by batch affects, which may influence our findings, particularly as there is significant overlap between batch and the study groups (Table S1). However, there were no significant differences in alpha (Figure S4G-I, Figure S8F-H) or beta diversity (Figure S3, S7) for neither mothers nor infants. Differential abundance analysis (Table S2-S7) however did detect some differences due to batch. Notably, most significant differences associated with batch did not overlap with those attributed to delivery mode or antibiotic use, or the change was in the opposite direction indicating our results are more likely to underreport the effect of delivery mode or antibiotics exposure. Hence, we do not think it is likely that the significant results we present in our research article are merely false positives because of batch effects.

Supplementary Figures

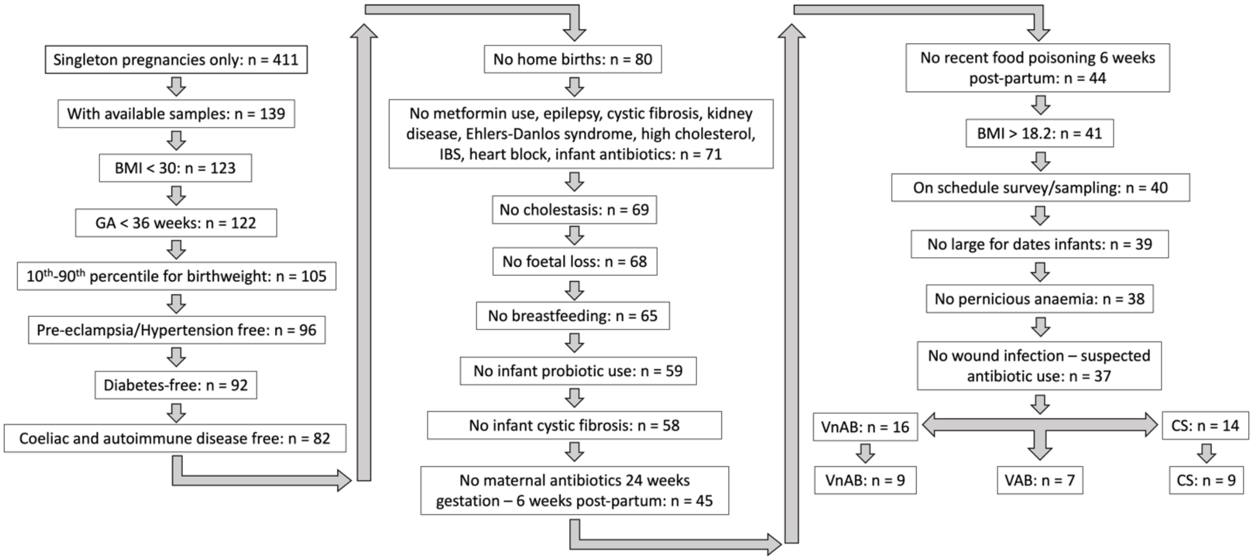

Figure S1: Exclusion of participants from original 411 singleton pregnancies included in the Queensland Family Cohort Pilot. A subset of available VnAB an CS dyads was taken to allow for approximately equal group sizes with approximately equal male and female infants.

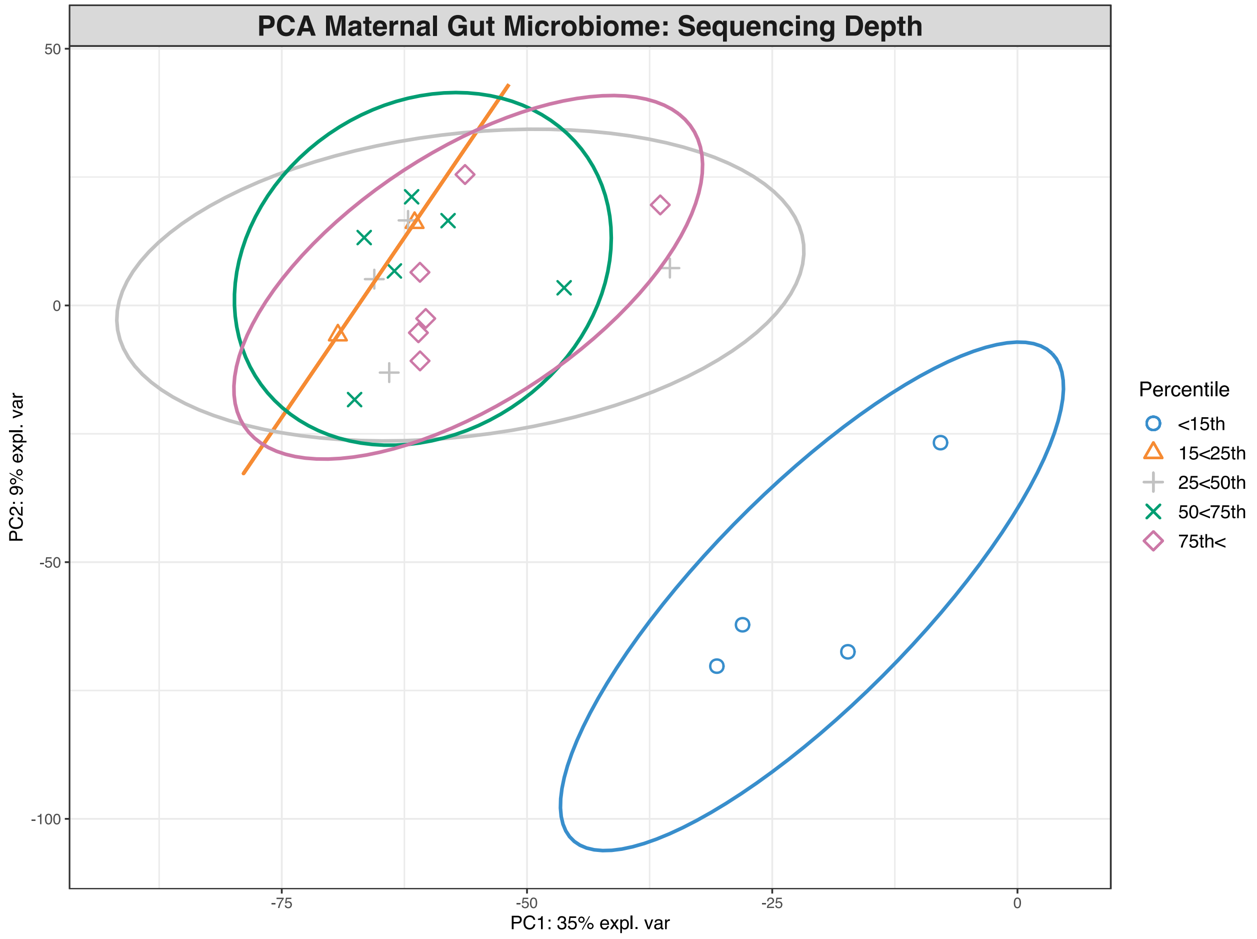

Figure S2: Principal component analysis of maternal gut microbiome prior to rarefaction and removal of low sequencing depth samples (<15th percentile) coloured by sequencing depth percentile category. Ellipses represent 95% confidence interval.

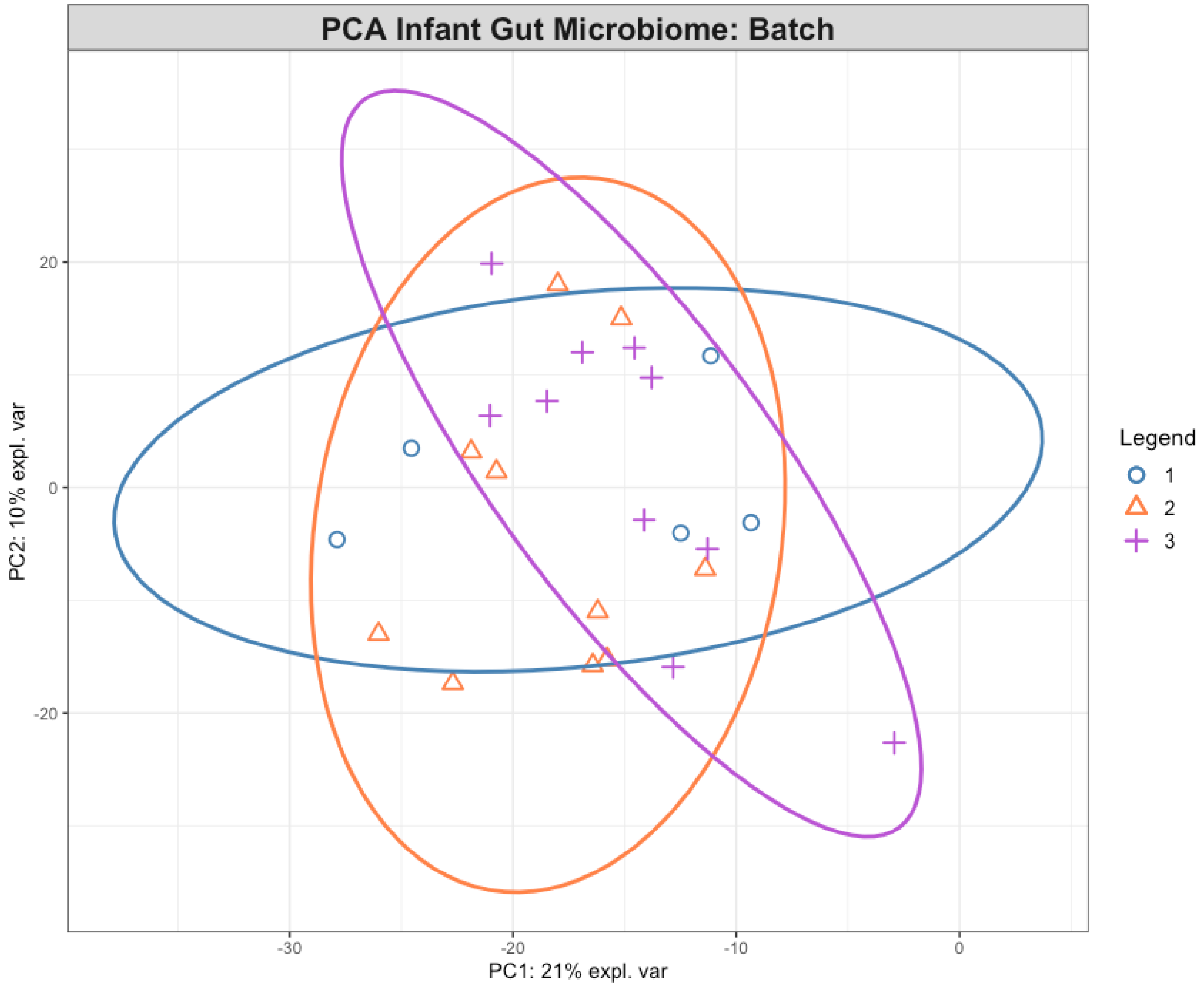

Figure S3: Principal component analysis of infant gut microbiome coloured by batch. Ellipses represent 95% confidence interval. Andonis2 $R^2 = 0.39$, $p = 0.52$.

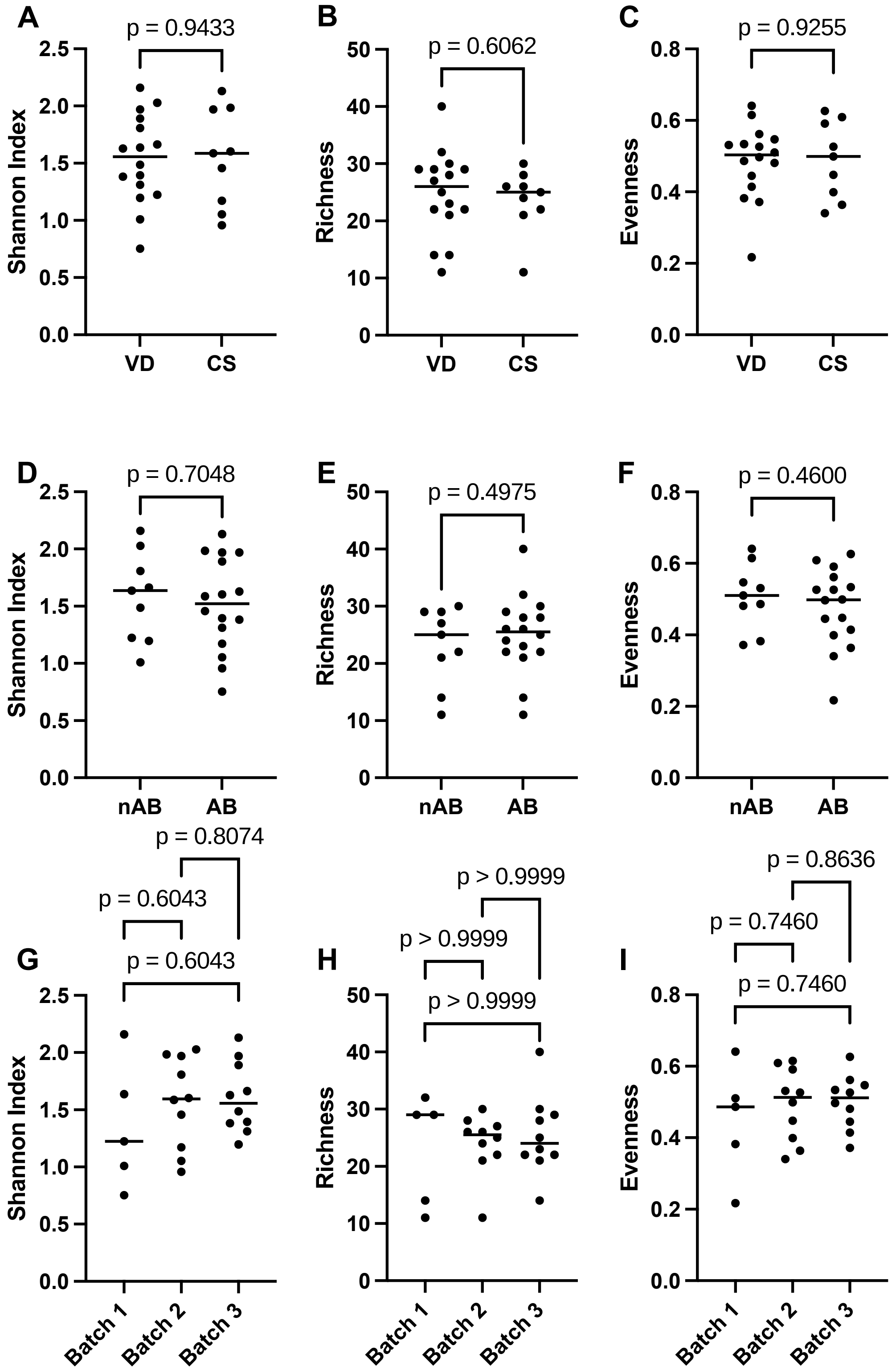

Figure S4: Alpha diversity of infant gut microbiome as measured by Shannon Index (**A,D,G**), Richness (**B,E,H**) and Evenness (**C,F,I**) by delivery mode (**A-C**), antibiotic use status (**D-F**) and batch (**G-I**). VD: Vaginal delivery, CS: Caesarean section, nAB: no antibiotic exposure during labour and delivery, AB: antibiotic exposure during labour and delivery

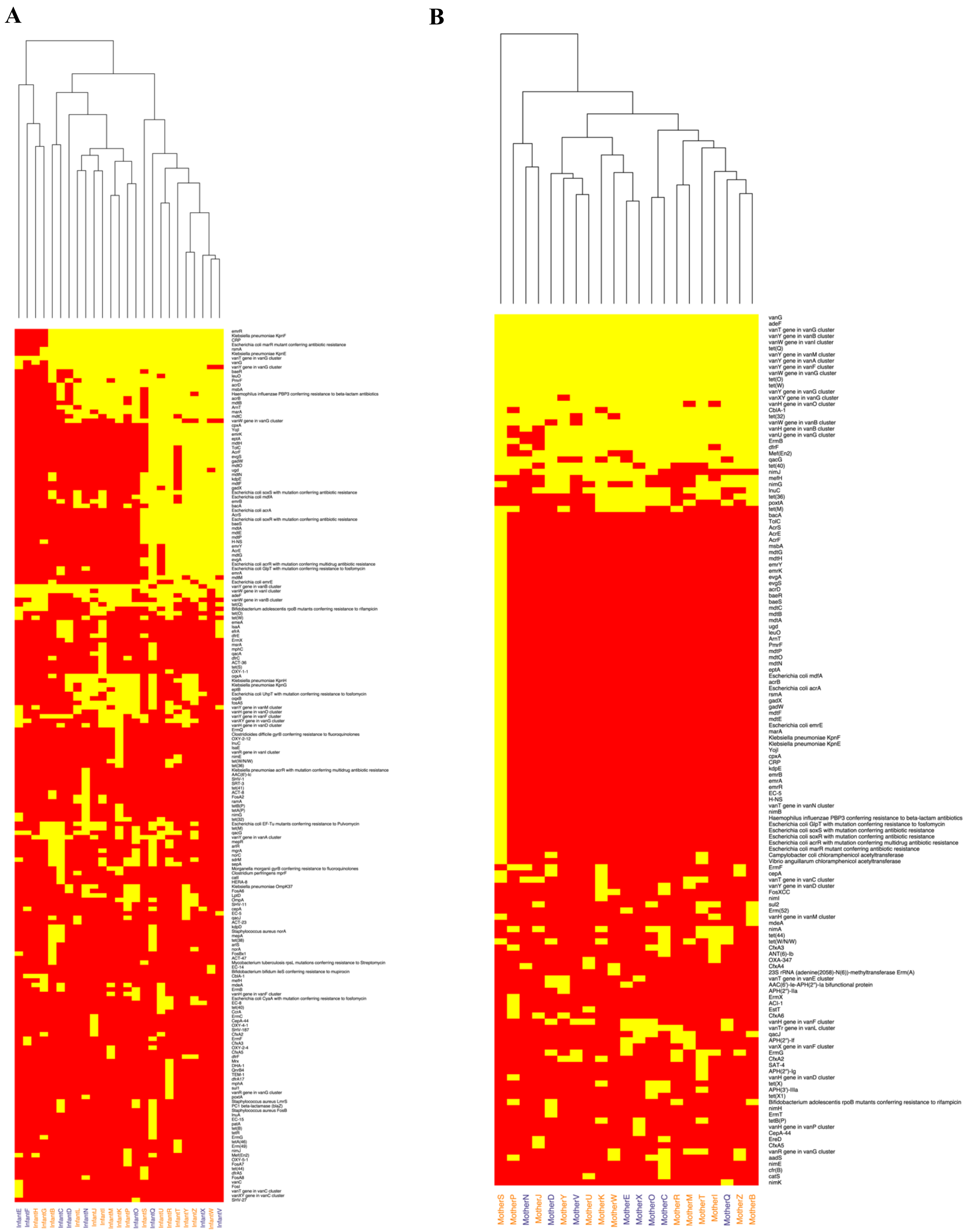

Figure S5: Presence/Absence (yellow/red) of antibiotic resistance genes in **A)** infant and **B)** maternal gut microbiota at 6 weeks post-partum. nAB in purple text and AB in orange text

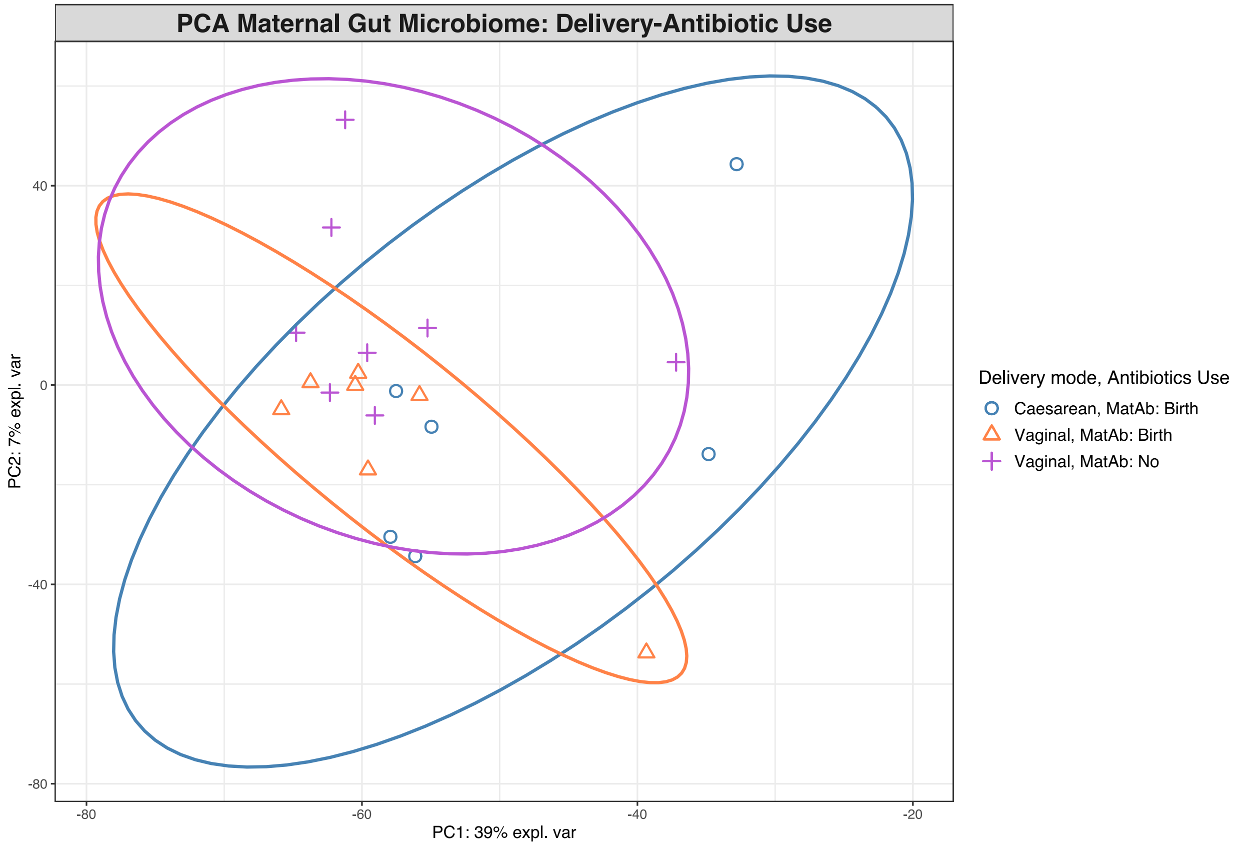

Figure S6: Principal component analysis of maternal gut microbiome coloured by delivery mode-antibiotic use status. Ellipses represent 95% confidence interval.

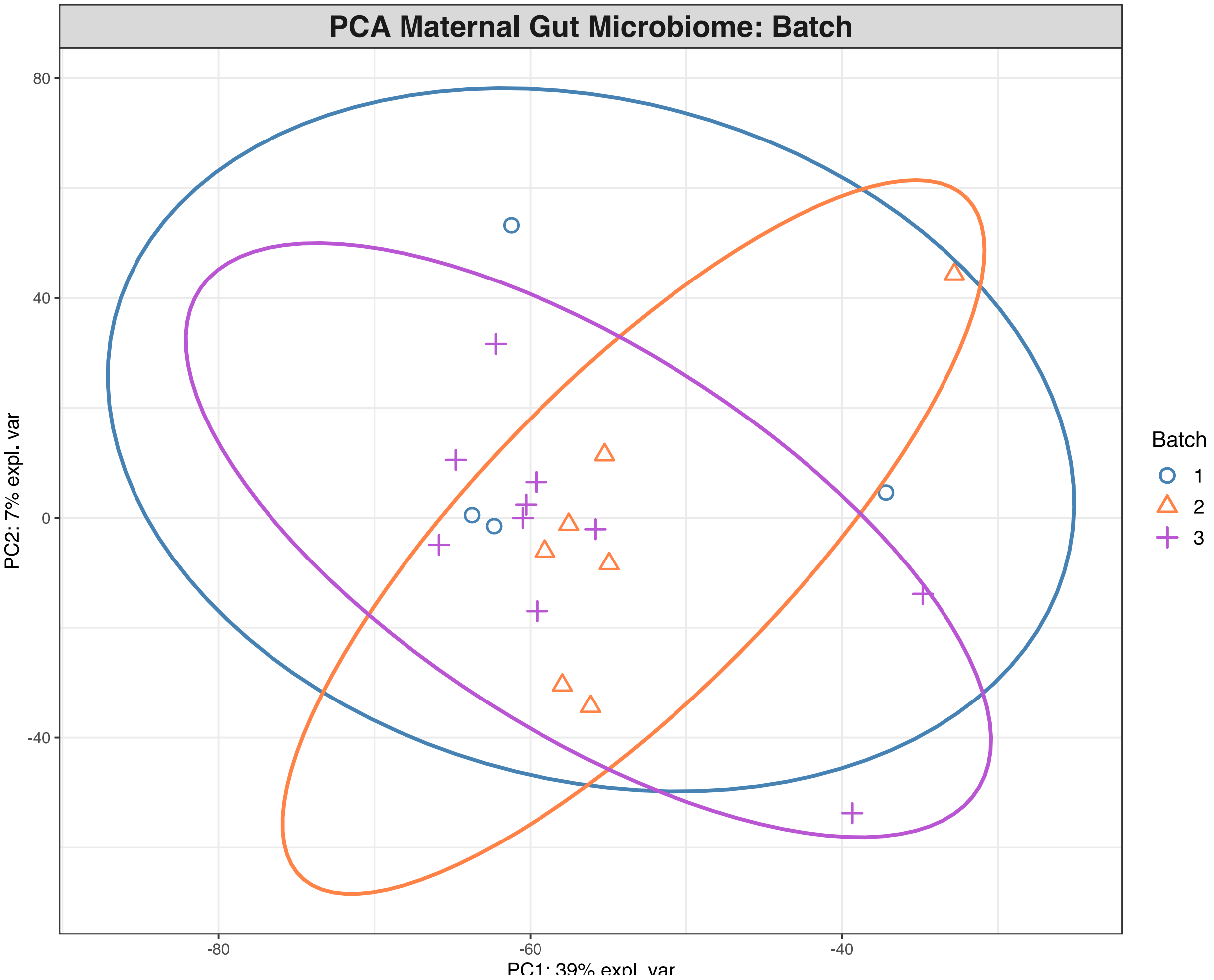

Figure S7: Principal component analysis of maternal gut microbiome coloured by batch. Ellipses represent 95% confidence interval.

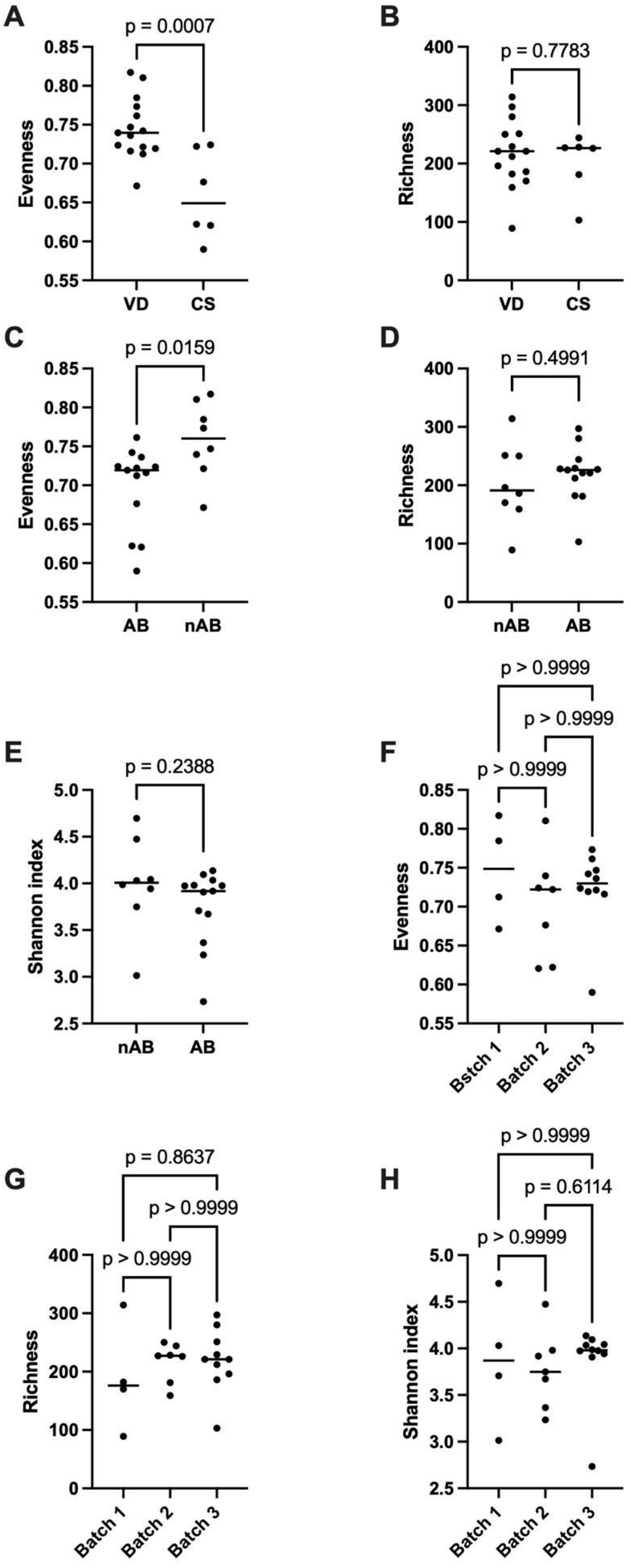

Figure S8: Alpha diversity of maternal gut microbiome as measured by Richness (**B,DG**), Shannon Index (**E,H**) and Evenness (**A,C,F**) by delivery mode (**A,B**), antibiotic use status (**C-E**) and batch (**F-H**). VD: Vaginal delivery, CS: Caesarean section, nAB: no antibiotic exposure during labour and delivery, AB: antibiotic exposure during labour and delivery

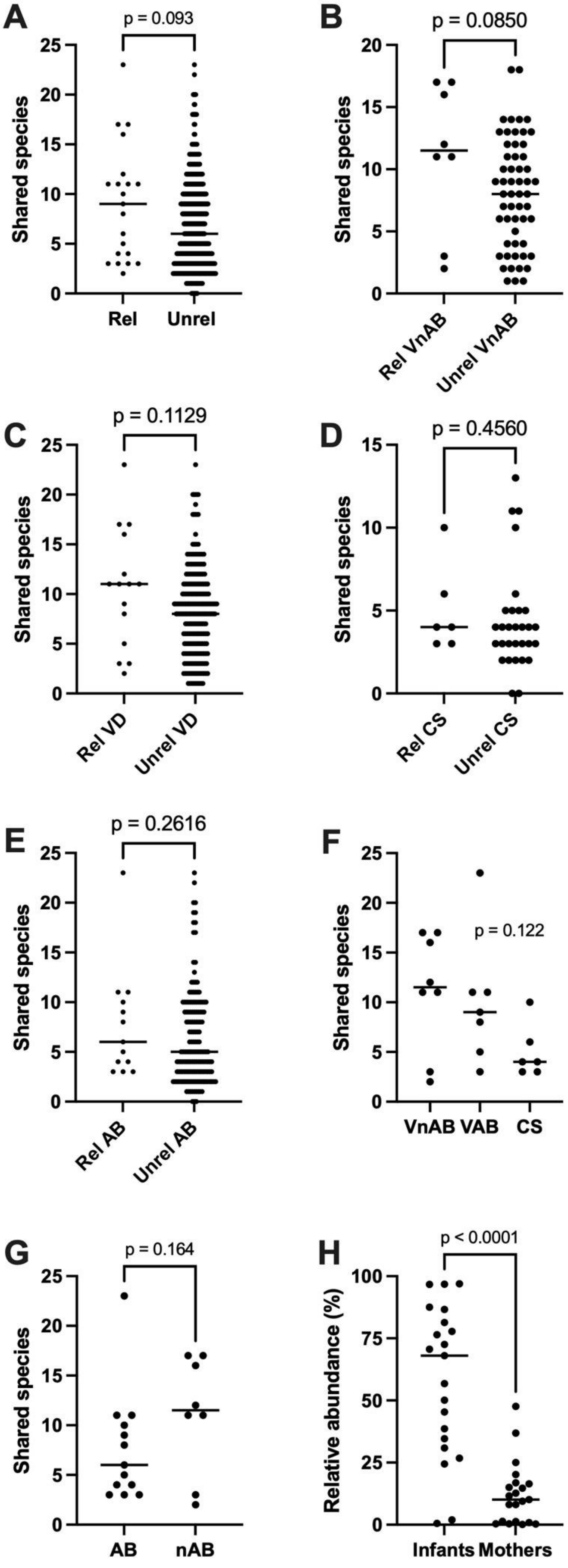

Figure S9: Comparison of shared species between **A)** all **B)** vaginally delivered, no antibiotic exposure, **C)** vaginally delivered, **D)** caesarean section, antibiotic exposed, and **E)** antibiotic exposed related and unrelated mother-infant dyads. Number of shared species by **F)** combined delivery mode-antibiotic use status, **G)** antibiotic use status. **H)** Relative abundance of shared species between related mother-infant pairs in infants and mothers

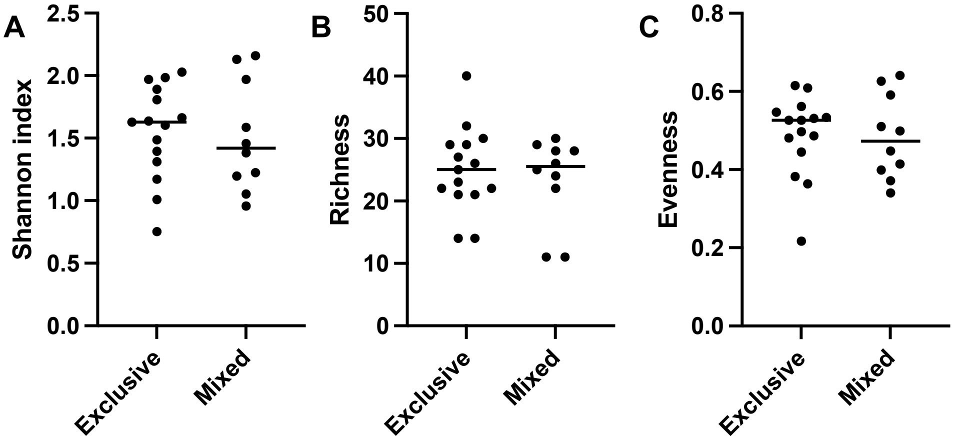

Figure S10: Effect of exclusive breastfeeding vs breastfeeding and formula (mixed) feeding on alpha diversity measured by **A)** Shannon index, **B)** Richness and **C)** Evenness

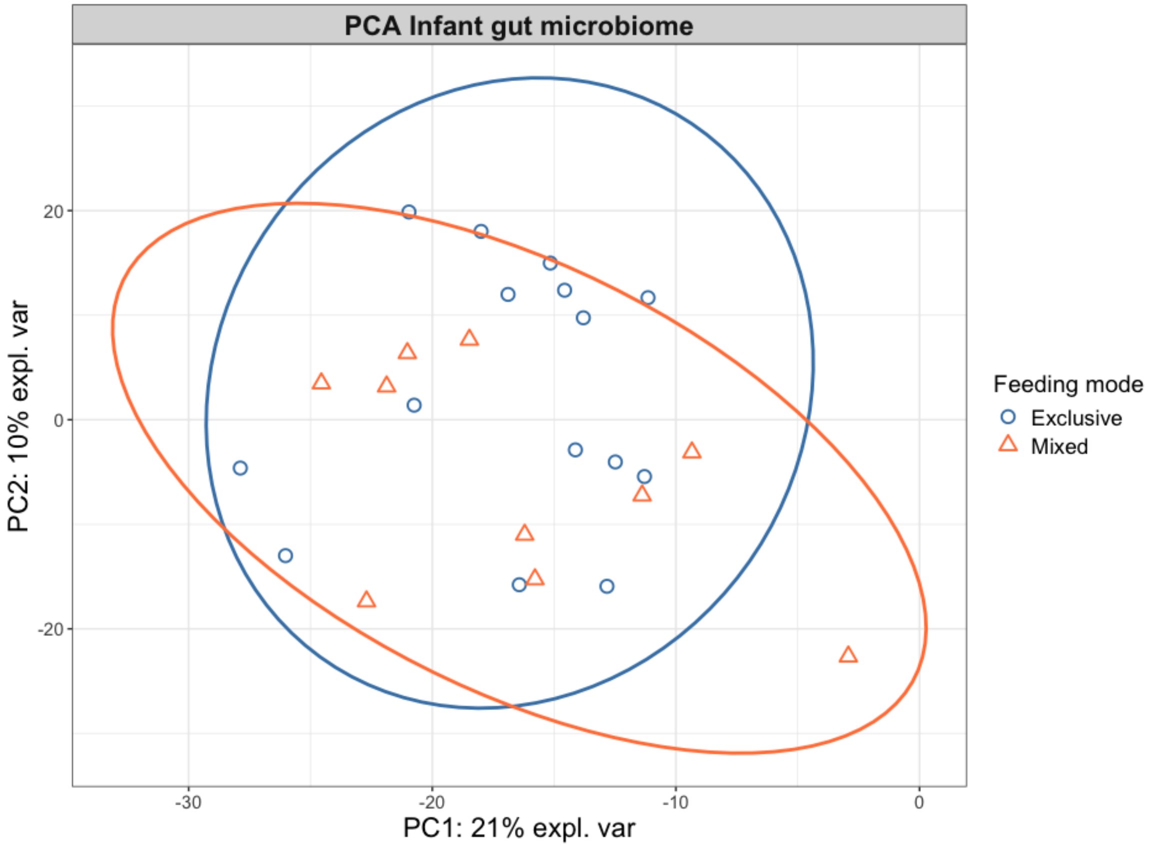

Figure S11: Principal component analysis of infant gut microbiome coloured by feeding mode (exclusive breastfeeding vs mixed breast and formula feeding). Ellipses represent 95% confidence interval.

Table S1: Additional Maternal Characteristics. nAB: no antibiotics during delivery, AB: antibiotics during delivery

	**Vaginal (nAB) (n=9)**	**Vaginal (AB) (n=7)**	**Caesarean (n=9)**	**p-value**
Retained Weight Gain (pre-pregnancy to 6 weeks post-partum) (kg)	7.1±3.9	4.7±4.9	6.0±3.6	0.53
**BATCH**				0.0005
Batch 1	4	1	0	
Batch 2	2	0	8	
Batch 3	3	6	1	
**ETHNICITY**				0.55
Caucasian	5	6	7	
Caucasian and Aboriginal Australian	1	0	0	
North-East Asian	2	0	1	
South/Central Asian	0	0	1	
South/Central American	1	1	0	
**REASON FOR ANTIBIOTICS**				0.0063
Prophylactic (Surgical)	-	1	9	0.0009
Premature rupture of membranes	-	5	0	0.0048
GBS positive	-	1	1	>0.99
Unknown	-	1	0	0.44
**CHRONIC DISEASE**				
Allergies/Dermatitis/Eczema	4	3	5	0.85
Mental health disorder	3	4	1	0.15
Endocrine disorder (inc. thyroid)	0	0	2	0.14
Ever diagnosed with Asthma	0	2	4	0.17
Endometriosis	1	0	1	0.66
Polycystic Ovary Syndrome (PCOS)	0	0	1	0.40
Osteoarthritis	0	0	1	0.40
Musculo-skeletal disease	1	1	0	0.53
Gilbert's syndrome	1	0	0	0.40
Sleep disorder	1	0	0	0.40
Blood coagulation defect	0	1	0	0.26
**DIET 6 weeks ##**				
Energy (kJ)	9073±2371	8961±1640	7381±1732	0.18
Carbohydrate (g/day)	238±76	244±61	190±48	0.21
Protein (g/day)	96±26	92±20	81±25	0.44
Fat (g/day) #	86 (74 – 98)	85 (73 – 100)	77 (60 – 92)	0.27
Saturated Fat (g/day)	31 (27 – 34)	37 (31 – 39)	27 (22 – 35)	0.11
Monounsaturated fat (g/day)	38±10	33±7	29±9	0.16
Polyunsaturated fat (g/day)	10 (9 – 16)	10 (8 – 13)	10 (7 – 12)	0.50
Dietary fibre (g/day)	31±11	28±8	26±9	0.49
Added sugar (g/day)	40±19	44±17	35±11	0.50
PHYSICAL ACTIVITY 6 weeks				
Sitting <8 hours/day	4	3^{6}	4^{8}	0.97
Meet activity guidelines	3	3^{6}	4	0.80

Data displayed as Mean ± SD, # Median (IQR), ^{4} n = 4 , ^{6} n = 6, ^{7} n = 7, ^{8} n = 8, * = significant pair-wise comparison

**Table S2:** Significant differentially abundant taxa (ANCOM-BC) in infants by delivery mode, antibiotics and batch. VD: Vaginal delivery, AB: antibiotics, B: Batch			
	Delivery mode	Antibiotics	Batch
Parabacteroides distasonis SGB 1934	↑ VD (q = 0.0003)	ns	ns
Bilophila wadsworthia SGB 15452	ns	ns	↓ B1 vs B2 (q = 0.001)
Lactobacillus gasseri SGB 7038	ns	↓ No AB (q = 0.025)	↑ B1 vs B3 (q = 0.02)
Clostridium tertium SGB 6183	↓ VD (q = 0.003)	ns	ns
Clostridium perfringens SGB 6191	↓ VD (q = 0.0009)	ns	ns
Dolosigranulum pigrum SGB 7017	ns	↑ No AB (q < 0.0001)	ns
Genus *Bacteroides*	↑ VD (q < 0.0001)	ns	ns
Genus *Phocaeicola*	↑ VD (q = 0.03)	ns	ns
Genus *Parabacteroides*	↑ VD (q = 0.0007)	ns	ns
Genus *Gemella*	↓ VD (q = 0.009)	ns	↑ B3 vs B2 (q < 0.0001)<br><br>↑ B1 vs B2 (q = 0.004)
Genus *Clostridium*	ns	ns	↑ B3 vs B2 (q = 0.0001)
Genus *Bilophila*	ns	ns	↓ B1 vs B2 (q = 0.04)
Genus *Dolosigranulum*	ns	↑ No AB (q < 0.0001)	ns
Haemophilus parainfluenzae SGB 9712	ns	ns	↓ B1 vs B2 (q = 0.0009)
Bifidobacterium breve SGB 17247	ns	ns	↓ B3 vs B2 (q < 0.0001)
Genus *Actinomyces*	ns	ns	↑ B3 vs B2 (q = 0.0006)
Genus *Flavonifractor*	ns	ns	↑ B3 vs B2 (q = 0.01)
Family *Bacteroidaceae*	↑ VD (q < 0.0001)	ns	ns
Family *Bacteroidales* unclassified	↑ VD (q = 0.02	ns	ns
Family *Tannerellaceae*	↑ VD (q = 0.0004)	ns	ns
Family *Bacillales* unclassified	↓ VD (q = 0.003)	ns	ns
Family *Desulfovibrionaceae*	ns	ns	↓ B1 vs B2 (q = 0.002)
Family *Carnobacteriaceae*	ns	↑ No AB (q = 0.01)	ns
Family *Pasteurellaceae*	ns	ns	↓ B1 vs B2 (q = 0.02)

Order *Bacteroidales*	↑ VD ($q < 0.0001$)	ns	ns
Order *Desulfovibrionales*	ns	ns	↓ B1 vs B2 ($q = 0.0006$)
Order *Pasteurellales*	ns	ns	↓ B1 vs B2 ($q = 0.003$)
Class *Bacteroidia*	↑ VD ($q < 0.0001$)	ns	ns
Class *Deltaproteobacteria*	↑ VD ($q = 0.004$)	ns	↓ B1 vs B2 ($q < 0.0001$)
Phylum *Bacteroidetes*	↑ VD ($q < 0.0001$)	ns	ns

Table S3: Significant differentially functional pathways (ANCOM-BC) in infants by delivery mode and batch. VD: Vaginal delivery, B: Batch

	Delivery mode	Batch
GALACT-GLUCUROCAT-PWY: superpathway of hexuronide and hexuronate degradation	↑ VD ($q = 0.0007$)	↓ B3 vs B2 ($q = 0.01$)
GLUCUROCAT-PWY: superpathway of β-D-glucuronosides degradation	↑ VD ($q = 0.001$)	ns
GLUDEG-I-PWY: GABA shunt I	↑ VD ($q = 0.01$)	ns
PWY-7456: β-(1,4)-mannan degradation	↑ VD ($q < 0.0001$)	ns
PWY0-42: 2-methylcitrate cycle I	ns	↓ B3 vs B2 ($q = 0.0005$)
PWY66-391: fatty acid β-oxidation VI	ns	↑ B3 vs B2 ($q < 0.0001$)
ARGDEG-PWY: superpathway of L-arginine, putrescine, and 4-aminobutanoate degradation	ns	↓ B3 vs B2 ($q < 0.0001$)
DARABCATK12-PWY: D-arabinose degradation II	ns	↓ B3 vs B2 ($q = 0.02$)
GALACTITOLCAT-PWY: galactitol degradation	ns	↓ B3 vs B2 ($q < 0.0001$)
HEXITOLDEGSUPER-PWY: superpathway of hexitol degradation (bacteria)	ns	↓ B3 vs B2 ($q < 0.0001$)
KETOGLUCONMET-PWY: ketogluconate metabolism	ns	↓ B3 vs B2 ($q = 0.0089$)
ORNARGDEG-PWY: superpathway of L-arginine and L-ornithine degradation	ns	↓ B3 vs B2 ($q < 0.0001$)
PWY-6531: mannitol cycle	ns	↓ B3 vs B2 ($q = 0.007$)
PWY-6922: L-Nδ-acetylornithine biosynthesis	ns	↓ B3 vs B2 ($q = 0.014$)
PWY-7209: superpathway of pyrimidine ribonucleosides degradation	ns	↓ B3 vs B2 ($q = 0.045$)
PWY-7340: 9-cis, 11-trans-octadecadienoyl-CoA degradation (isomerase-dependent, yeast)	ns	↑ B3 vs B2 ($q < 0.0001$)

Table S6: Significant differentially abundant functional pathways (ANCOM-BC) in mothers by delivery mode and antibiotics, with effect of batch. VD: Vaginal delivery, AB: antibiotics, B: Batch.

	Delivery mode	Antibiotics	Batch
METHGLYUT-PWY: superpathway of methylglyoxal degradation	ns	↓ No AB (*q* = 0.001)	↓ B3 vs B2 (*q* = 0.0016)
PWY-6906: chitin derivatives degradation	↑ VD (*q* = 0.01)	ns	ns
PWY-7315: dTDP-N-acetylthomosamine biosynthesis	↑ VD (*q* < 0.0001)	ns	↓ B3 vs B2 (*q* = 0.04)
PWY490-3: nitrate reduction VI	ns	↑ No AB (*q* = 0.02)	ns
GALACT-GLUCUROCAT-PWY: superpathway of hexuronide and hexuronate degradation	ns	ns	↓ B3 vs B2 (*q* < 0.0001)<br>↑ B1 vs B3 (q = 0.001)
GALACTUROCAT-PWY: D-galacturonate degradation I	ns	ns	↓ B3 vs B2 (*q* < 0.0001)
P125-PWY: superpathway of (R,R)-butanediol biosynthesis	ns	ns	↑ B3 vs B2 (*q* = 0.02)
PWY-6507: 4-deoxy-L-threo-hex-4-enopyranuronate degradation	ns	ns	↓ B3 vs B2 (*q* = 0.03)<br>↑ B1 vs B3 (q = 0.003)
PWY-7013: (S)-propane-1,2-diol degradation	ns	ns	↑ B3 vs B2 (*q* = 0.03)
PWY0-1261: peptidoglycan recycling I	ns	ns	↓ B3 vs B2 (*q* = 0.003)
PWY66-389: phytol degradation	ns	ns	↑ B3 vs B2 (*q* < 0.0001)
P221-PWY: octane oxidation	ns	ns	↑ B1 vs B2 (*q* < 0.0004)
PWY-5265: peptidoglycan biosynthesis II (staphylococci)	ns	ns	↓ B1 vs B2 (*q* < 0.0001)
PWY-6478: GDP-D-glycero-α-D-manno-heptose biosynthesis	ns	ns	↓ B1 vs B2 (*q* < 0.0001)
PWY0-42: 2-methylcitrate cycle I	ns	ns	↑ B1 vs B2 (*q* = 0.01)
